# Prenatal ultrasound diagnosis of ectopic ureter and renal hypoplasia in two puppies: a case report

**DOI:** 10.1007/s11259-025-10732-w

**Published:** 2025-04-11

**Authors:** H. Moserová, L. Frgelecová, R. Morávek, P. Proks

**Affiliations:** 1 Department of Diagnostic Imaging, Faculty of Veterinary Medicine, Small Animal Clinic, University of Veterinary Sciences Brno, Brno, 612 42 Czech Republic; 2 Department of Pathological Morphology and Parasitology, University of Veterinary Sciences Brno, Brno, 612 42 Czech Republic

**Keywords:** Ultrasound, Prenatal diagnosis, Ectopic ureter, Renal hypoplasia

## Abstract

This study reports two cases of prenatal ultrasound diagnosis of ectopic ureter and bilateral renal hypoplasia in two different canine fetuses. These developmental disorders were detected during a routine pregnancy ultrasound examination of a female Golden retriever and a female Old English bulldog. A suspected diagnosis of ectopic ureter was made based on the ultrasonographic detection of fetal kidney hydronephrosis and ureteral dilatation, along with consideration of breed predisposition. Bilateral fetal renal hypoplasia presented with ultrasonographic detection of reduced, hyperechogenic fetal kidneys without a distinguishable renal pelvis. Ultrasonographic findings were subsequently confirmed postnatally at surgery or autopsy and histopathology. Our findings highlight the potential for early detection of urogenital anomalies in canines and the importance of knowledge of normal fetal anatomy during ultrasound examination of pregnancy.

## Background

Ultrasound imaging has emerged as a valuable diagnostic tool in theriogenology, offering a non-invasive, cheap and widely available means of assessing fetal development during pregnancy. Due to the development in ultrasonographic technology, studies detailing normal prenatal anatomy of various organ systems in dogs including kidneys have been published (Gil et al. 2017; Maronezi et al. 2022; Siena et al. 2022). In the late pregnancy, canine fetal kidney length, cortical and medullary thickness and cortico-medullary thickness ratio are proportional to the number of days before parturition, with the kidney length and cortical thickness being influenced by maternal size (Siena et al. 2022). The improving level of knowledge about fetal kidney anatomy allows for early diagnosis of congenital developmental defects, similar to human medicine, where renal anomalies are among the most frequent prenatally diagnosed fetal disorders (Dias et al. 2014). To the authors’ knowledge, this is the first documentation of ultrasonographic prenatal diagnosis of ectopic ureter and prenatal diagnosis of bilateral renal hypoplasia in canine fetuses.

## Case presentation

### Case 1

**Fig. 1:**
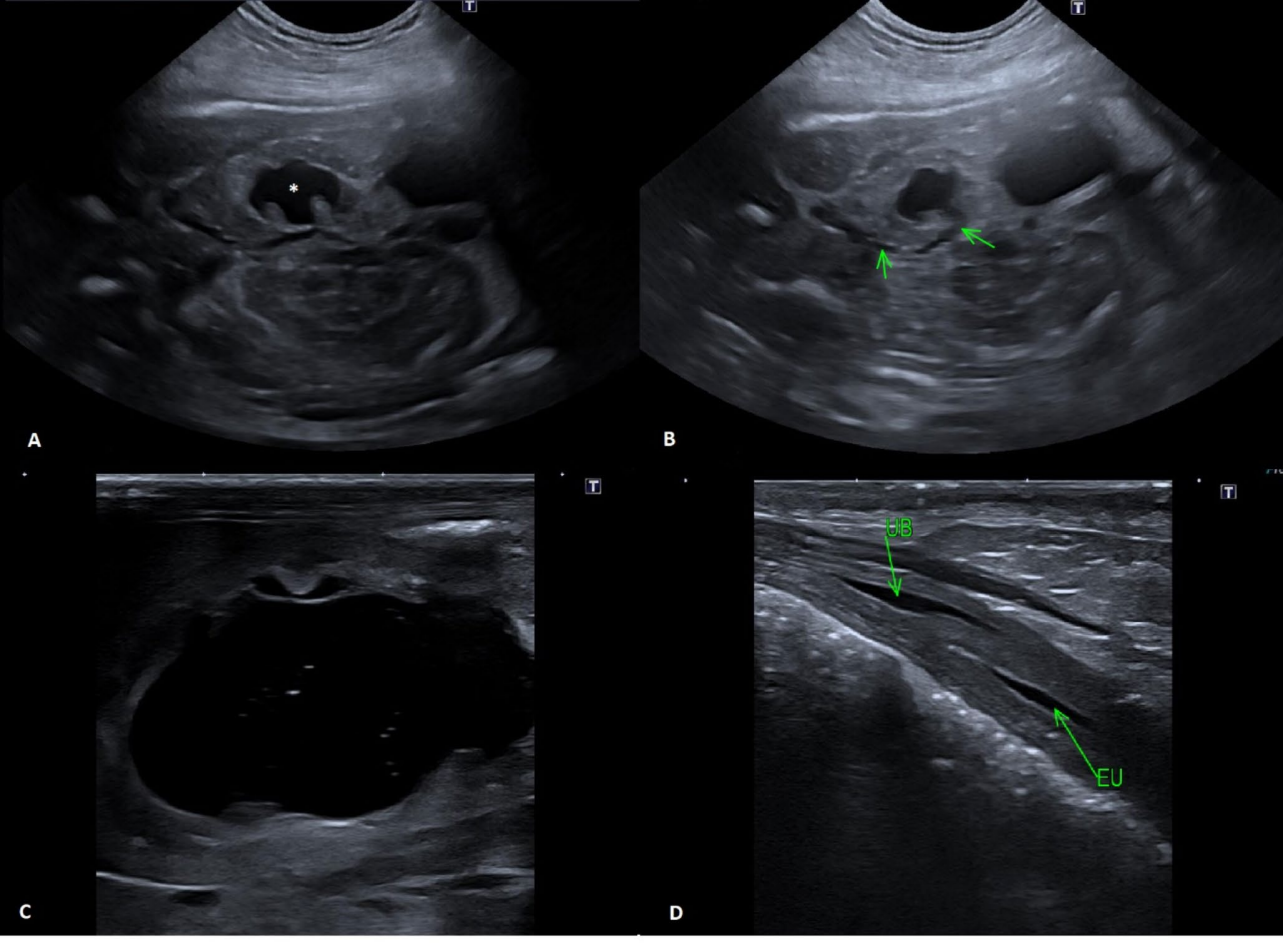
**A** The left fetal kidney with a markedly dilated renal pelvis (asterisk) during prenatal ultrasound examination with a 11 MHz microconvex transducer. **B** Prenatal ultrasound with a 11 MHz microconvex transducer. The fetal ureter, mildly dilated in its course, is marked with arrows. **C** The hydronephrotic left kidney during postnatal examination of a female puppy with a 22 MHz linear transducer. **D** Postnatal examination of a female puppy with a 22 MHz linear transducer. The ectopic ureter (EU) is seen in the longitudinal plane, coursing caudally from the urinary bladder neck (UB) separated by a thick wall

 A six-years-old clinically healthy pregnant female Golden retriever, body weight 40 kg, was presented to the department of diagnostic imaging of the University of Veterinary Sciences Brno (VETUNI) 56 days after ovulation for fetal count radiography and ultrasound evaluation of fetal viability. One right latero-lateral view of the abdomen was obtained, with the detection of six fetuses with normal radiographic morphology. The ultrasound examination was performed using a Canon Aplio i700 (Canon Medical Systems Corporation, Tochigi, Japan) ultrasound machine. All fetuses were viable on ultrasound with heart rate in range of 220–240 beats per minute, as measured with M-mode technique. During the examination of the first fetus, marked left renal pelvis dilation with anechoic content was noted (Fig. 1A). The measured size of the left renal pelvis was 13.2 × 9.4 mm in the longitudinal plane. Furthermore, we detected the course of the left ureter, which was also slightly dilated to a width of 1–1.7 mm (Fig. 1B). The dilated left ureter coursed caudally to the pelvic inlet area, where it could no longer be traced. Given the breed predisposition, the suspicion of ectopic ureter was raised, even though both parents were clinically healthy according to owner.

The female was presented again on 64 th day after ovulation due to labor difficulties. Ultrasound revealed one dead fetus positioned at the pelvic inlet, and one viable fetus positioned more cranially with a heart rate of 220 beats per minute. The owner decided for a cesarean section, during which one live and one dead fetus were delivered. The owner also brought the remaining four naturally born puppies and requested a sonographic kidney screening to identify the puppy with a suspected ectopic ureter. After thorough disinfection of the ultrasound machine and surfaces, a swift examination of the puppies was performed without hair clipping. A hydronephrotic left kidney was identified in the second-born female puppy (Fig. 1C), with the renal pelvis measuring 23 × 13 mm in longitudinal plane at that time. The left ureter (Fig. 1D) was dilated in the entire course to a width of 2–6 mm with a suspected ureteral opening into the pelvic urethra. A presumptive diagnosis of left extramural ectopic ureter was made based on the ultrasound findings. Subsequently, the owner confirmed permanent incontinence of this puppy a few days after birth via e-mail communication.

Surgical transposition was planned for after the puppy was 10 weeks old. However, the health condition was complicated by a severe bacterial infection of the urinary tract at 6 weeks of age. For this reason, the transposition was performed earlier, at 8 weeks of age. Surgery confirmed the presence of a left extramural ectopic ureter with ureteral opening into the pelvic urethra. After the transposition and treatment of UTI, the mild signs of urinary incontinence persisted for six weeks, especially during the night, but slowly improved. On the follow-up two months after the surgery, the patient was clinically healthy according to owner.

### Case 2

A three-years-old clinically healthy pregnant female Old English bulldog, body weight 22 kg, was presented to the department of diagnostic imaging of VETUNI 54 days after ovulation for fetal count radiography, ultrasound evaluation of fetal viability and planning of an elective cesarean section. One right latero-lateral view of the abdomeny was obtained, with the detection of nine fetuses with normal radiographic morphology. The ultrasound examination was performed using a Canon Aplio i700 ultrasound machine as in first case. All fetuses were viable on ultrasound with heart rate in range of 220–240 beats per minute, as measured with M-mode technique. The biparietal diameter, radius length and kidney length were measured to determine the parturition date.

**Fig. 2:**
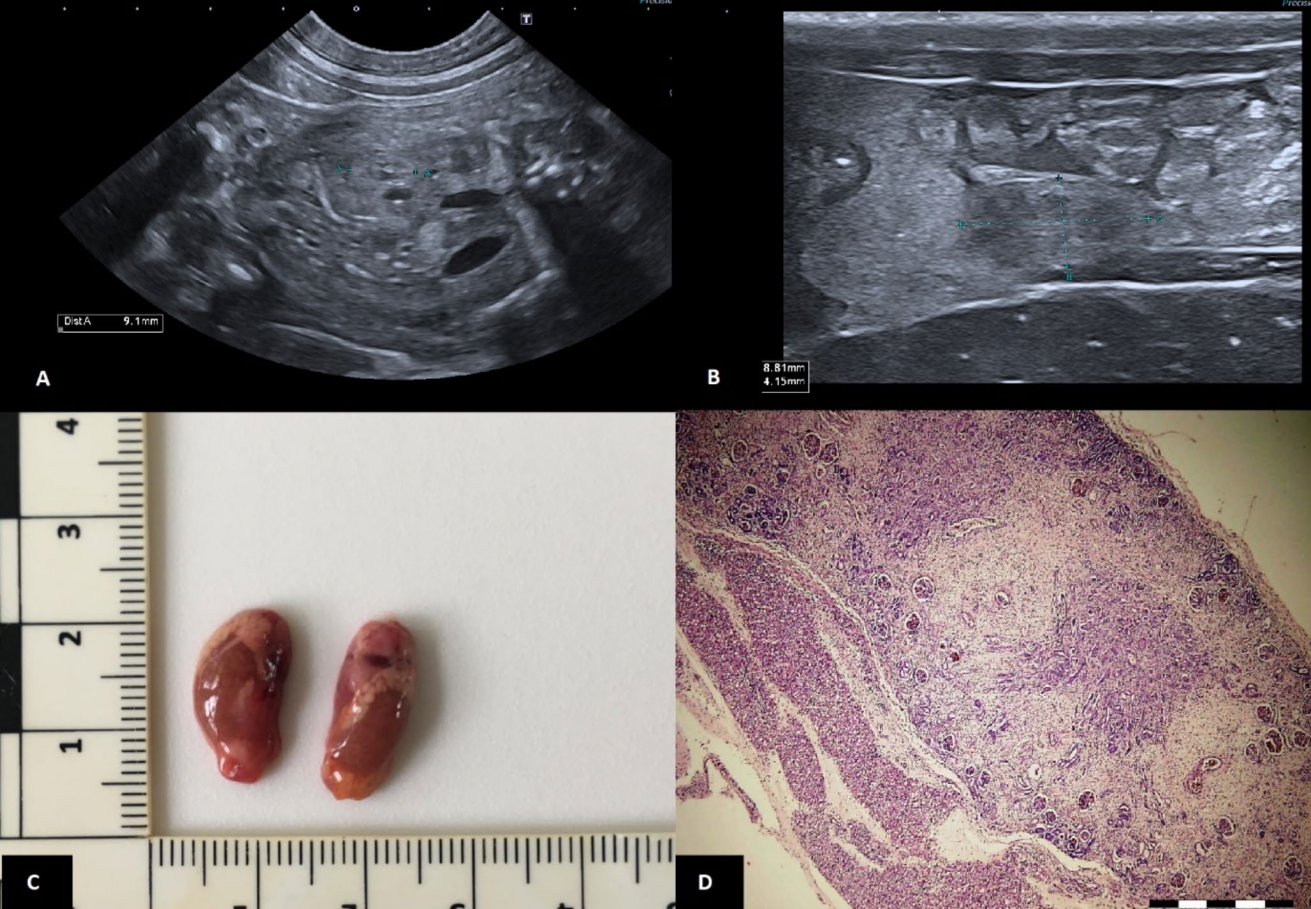
**A** Prenatal ultrasound measurement of the hypoplastic right fetal kidney (between the calipers) with a 11 MHz microconvex transducer. The kidney is reduced in size, generally hyperechogenic with an indistinguishable renal pelvis. **B** Postnatal ultrasound measurement of the hypoplastic left kidney (between the calipers) with a 22 MHz linear transducer. **C** Hypoplastic kidneys at the autopsy, with attached ruler indicating the macroscopic kidney size. **D** Histopathologic image of the kidney showing undifferentiated parenchyma in the majority of the kidney. Though the differentiation into glomerular and tubular structures is evident in some parts, most of the parenchyma is replaced by connective tissue. The hematoxylin-eosin staining was used

During the measurement, very small and hyperechoic kidneys were found in one of the fetuses (Fig. 2A). The measured length of both kidneys was approximately 9 mm; kidneys of other fetuses were around 20–22 mm in length for comparison. Renal pelvis of the reduced kidneys was indistinct, even though it was visible in the kidneys of other fetuses. Based on these findings, bilateral renal hypoplasia was suspected in this fetus.

During the cesarean section, one of the puppies was smaller and its amniotic fluid had uremic scent. The puppy showed only weak signs of life and died approximately an hour after birth despite the life support. The owner agreed to an ultrasound examination and subsequent autopsy to identify the cause of death. Post-mortem ultrasound confirmed that it was indeed the fetus with small, hyperechogenic kidneys (Fig. 2B). This finding was consistent with the autopsy, where the kidneys were significantly reduced in size (Fig. 2C). No additional pathologies were detected during autopsy. Histologic examination revealed marked hypoplastic and dysplastic changes of both kidneys (Fig. 2D).

## Discussion and conclusion

In the presented cases, prenatal ultrasound examination was effective in diagnosing renal congenital abnormalities in two canine fetuses. Prenatal diagnostics is a growing trend in veterinary medicine, with ultrasound prenatal diagnoses of hydrocephalus, fetal anasarca, interstitial nephritis, abdominal wall and diaphragmatic hernia, gastrointestinal obstruction and other disorders already documented (Lopate 2023; Sananmuang et al. 2019; Siena et al. 2021; Silva et al. 2021).

On ultrasound, the canine fetal kidneys are first visualized around 37–45 days post-ovulation (Yeager et al. 1992). On day 24 − 20 before parturition, they are “mushroom-shaped” with a distinct dilated renal pelvis. Renal cortex and medulla are ultrasonographically distinguishable from day 43 of pregnancy (Gil et al. 2017). The dilatation of the renal pelvis decreases as the pregnancy advances, and physiologically disappears after 57 days of gestation; the kidney from this point onwards resembles that of a healthy adult (Gil et al. 2017).

The finding of a significantly dilated renal pelvis and ureter in a late pregnancy, together with the known breed predisposition (Holt and Moore 1995; Holt et al. 2000), led to the suspected diagnosis of ectopic ureter in the first case. Ureteral ectopia is often associated with hydroureter and hydronephrosis, presumably due to the abnormality creating back pressure of urine leading to altered ureteral peristalsis (Holt and Moore 1995). Although operator-dependent, ultrasound has already proven to be a suitable modality for screening adult dogs for ureteral ectopia prior to cystoscopy, achieving sensitivities of 87.8–93.6% and specificities of 86.2–100% (Kendall et al. 2024; Taylor et al. 2022). Based on our findings, the diagnosis of ectopic ureter should be considered in cases of dilatation of the renal pelvis and ureter during prenatal ultrasound examination in predisposed breeds. However, other congenital or developmental diseases such as interstitial nephritis or ureteral stenosis may present with similar ultrasound findings and are therefore important differential diagnoses (Pullium et al. 2000; Silva et al. 2021). Prospective studies with a larger number of dogs would be necessary to evaluate the sensitivity and specificity of prenatal ultrasound examination in ectopic ureter diagnostics.

On fetal or postnatal ultrasound in human medicine, renal hypoplasia is defined as a kidney volume of below two standard deviations of a mean kidney volume for the given age (Cain et al. 2010). The kidneys of the fetus in the second case in this report were very small compared to the kidneys of the other fetuses, differing by more than half the expected kidney length. The authors attribute the death of this fetus shortly after delivery to bilateral involvement. Bilateral renal hypoplasia leading to death is recorded in Large White piglets; the piglets would die at birth or within the first 3 months of life (Constable et al. 2017). In dogs, the reported clinical picture of renal hypoplasia includes electrolyte disbalances, uremia and anemia, identical to cases of chronic nephropathy (Persson et al. 1961). Though fatal in our case, unilateral renal hypoplasia may remain clinically undetectable except for compensatory hypertrophy and related enlargement of the healthy kidney (Constable et al. 2017).

In conclusion, ultrasonographic prenatal examination is of utmost importance in detecting abnormalities that may affect the course of parturition and the possible occurrence of dystocia as well as perinatal and postnatal fetal viability. Knowledge of the physiological ultrasound morphology at a given stage of development, along with comparison of ultrasound findings in multiple fetuses, appear to be essential in prenatal diagnosis of developmental disorders in dogs. Early detection of developmental renal disorders is important for the prediction of perinatal survival, and applying adequate care during and after delivery can minimize the risk of permanent damage or loss of organ function.

## Data Availability

No datasets were generated or analysed during the current study.
